# Assessment of yeast *Saccharomyces cerevisiae* component binding to *Mycobacterium avium* subspecies *paratuberculosis* using bovine epithelial cells

**DOI:** 10.1186/s12917-016-0665-0

**Published:** 2016-03-01

**Authors:** Ziwei Li, Qiumei You, Faisury Ossa, Philip Mead, Margaret Quinton, Niel A. Karrow

**Affiliations:** Department of Animal Biosciences, University of Guelph, Guelph, N1G 2W1 ON Canada; Lallemand Inc., Montréal, H4P 2R2 QC Canada

**Keywords:** *Saccharomyces cerevisiae*, *Mycobacterium avium* spp. *paratuberculosis* (MAP), Epithelial cell adhesion

## Abstract

**Background:**

Since yeast *Saccharomyces cerevisiae* and its components are being used for the prevention and treatment of enteric diseases in different species, they may also be useful for preventing Johne’s disease, a chronic inflammatory bowel disease of ruminants caused by *Mycobacterium avium* spp. *paratuberculosis* (MAP). This study aimed to identify potential yeast derivatives that may be used to help prevent MAP infection. The adherence of mCherry-labeled MAP to bovine mammary epithelial cell line (MAC-T cells) and bovine primary epithelial cells (BECs) co-cultured with yeast cell wall components (CWCs) from four different yeast strains (A, B, C and D) and two forms of dead yeast from strain A was investigated.

**Results:**

The CWCs from all four yeast strains and the other two forms of dead yeast from strain A reduced MAP adhesion to MAC-T cells and BECs in a concentration-dependent manner after 6-h of exposure, with the dead yeast having the greatest effect.

**Conclusions:**

The following in vitro binding studies suggest that dead yeast and its’ CWCs may be useful for reducing risk of MAP infection.

## Background

Dairy producers have been using commercially available yeast probiotics and their components as feed supplements for nearly two decades based on claims that these products will improve animal production, promote health, and reduce the need for antibiotic use. Studies demonstrate that supplementing the ruminant diet with specific strains of *S. cerevisiae* improves feed intake [1, 2], weight gain [3], and fiber digestion [4, 5]. It has also been reported that live yeast stabilizes rumen pH [6, 7], and the number of anaerobic cellulolytic bacteria [8, 9]. In addition to having nutritional value, there is evidence that yeast probiotics and their components, such as mannan-oligosaccharides (MOS), can adhere to enteric pathogens including *Campylobacter jejuni* [10], *Escherichia coli* serotypes (O2 and O88) [11], and *Salmonella* [12, 13], thereby reducing their ability to attach to and invade host cells [14]. Given these properties, it is possible that dietary supplementation with yeast probiotics and/or their components may help protect calves that are vulnerable to Johne’s disease (JD).

Neonatal calves and calves less than six months of age are most susceptible to MAP infection, the causative agent of JD [15, 16], in part, due to their under-developed immune system [17]. MAP invades and subsequently damages the small intestine, causing diarrhea, weight loss and severe dehydration, and also reduces milk production [18]. Clinical signs of JD may take 2 to 5 years to develop [19], and its long subclinical stage facilitates continuous exposure of non-infected animals within an infected herd. MAP has a thick lipid-rich cell wall containing mycolic acids, which in part contributes to its resilience to chemical and physical degradation [20–22]. Currently, there is no satisfactory treatment for JD, since antibiotics only help to contain the disease, and vaccines only help to reduce disease incidence but they do not eliminate the disease completely [23]. Presently, the best way to control JD is through good management practices and early detection using different commercial available diagnostic tests [24], however, these diagnostic tests all have limitations and are costly.

Given these limitations, alternative strategies need to be explored to help reduce calf exposure to MAP and stimulate the host immune system to help combat MAP infection. We hypothesized that yeast and its CWCs may help reduce MAP adhesion to bovine epithelial cells, thereby reducing the risk of JD.

## Methods

### MAP isolation

The mCherry-labeled MAP used in the present study was developed by Mead [25] using the clinical isolate Gc86 strain previously isolated in the laboratory of Dr. Lucy Mutharia by Melinda Raymond (Department of Molecular and Cellular Biology, University of Guelph).

### Preparation of MAP infection stock

**Fig. 1:**
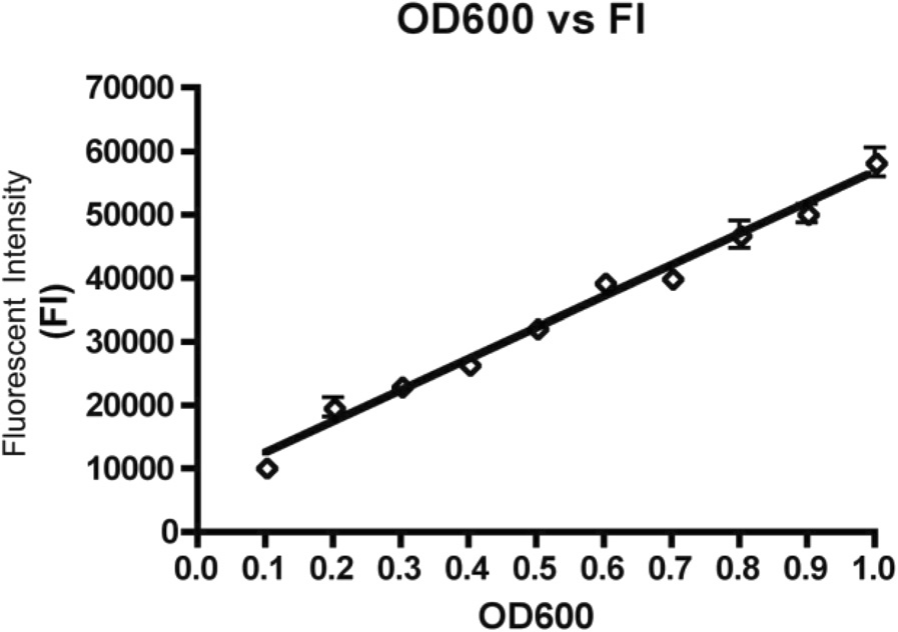
OD_600_ versus fluorescent intensity of mCherry MAP Gc86 [25]

**Fig. 2:**
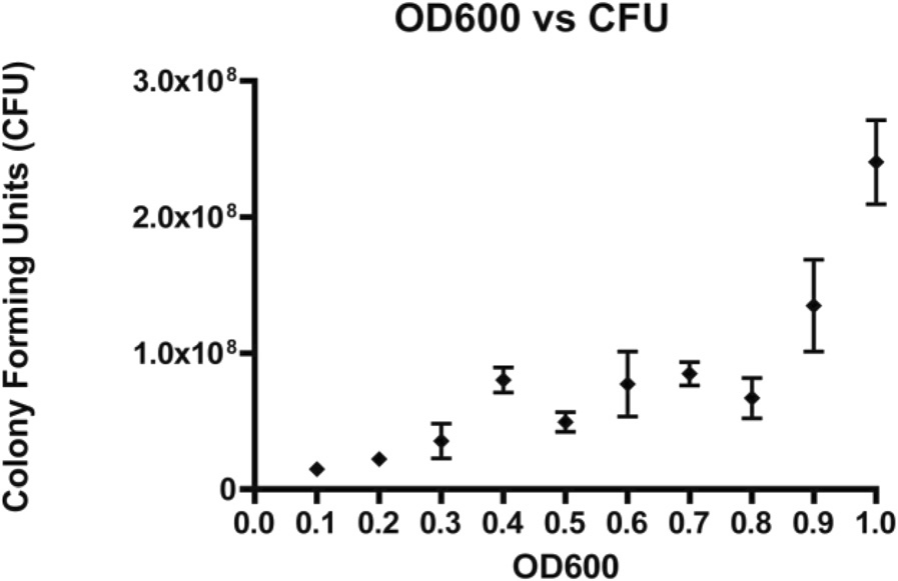
OD_600_ versus CFU of mCherry MAP Gc86 [25]

Liquid nitrogen frozen mCherry MAP Gc86 was thawed at 37 °C and was used to inoculate 5 ml of 7H9 broth (Difco laboratories, Franklin Lakes, NJ, USA) supplemented with 10 % oleic acid-albumin-dextrose-catalase (OADC; Sigma-Aldrich, St. Louis, MO, USA), 0.25 % v/v Tyloxopol (Sigma-Aldrich), 50 μg/ml kanamycin (Allied Laboratories, Wichita, KS, USA) and 2 μg/ml of mycobactin J (Allied Laboratories). The cultures were incubated at 37 °C, and once they reached a fluorescent intensity (FI) of 45000, equivalent to OD_600_ = 0.8 [25], 5 ml aliquots were sub-cultured into 100 ml of media in a 250 ml sterile culture flask and incubated at 37 °C. When the cultures reached the logarithmic stage of growth (FI = 40000–50000 equivalent to OD_600_ = 0.6–0.9), cells were centrifuged at 2000 × g for 30 min. The cells were re-suspended to reach FI = 60000 equivalent to OD_600_ = 1.0 using Fig. 1, then to establish Colony Forming Units (CFU/ml) using Fig. 2 [25]. Quantification of fluorescence was based on the specific emission (587 nm) and excitation wavelengths (610 nm) for mCherry using the Wallac-1420 VICTOR3 Multilabel Counter (Perkin Elmer, Woodbridge, ON, Canada).

### Bovine mammary epithelial cell line (MAC-T cells) and culture conditions

The MAC-T cells were cultured according to the reference [26]. MAC-T cells were cultured in T75 tissue culture flasks (Corning, Tewksbury, MA, USA) at 37 **°**C with 5 % CO_2_, containing Dulbecco's Modified Eagle Medium (DMEM; Invitrogen, Burlington, ON, Canada) supplemented with 4.0 mM L-glutamine, 10 % heat inactivated fetal bovine serum (FBS; Invitrogen), 25 mM HEPES buffer (Invitrogen), 0.25 μg/ml amphotericin B (Invitrogen), 1 % Penicillin/Streptomycin (100 unit/ml of Penicillin and 100 μg/ml Streptomycin; Invitrogen), 1 mM Sodium Pyruvate (Invitrogen), and 5 μg/ml insulin-transferrin-selenium (Invitrogen).

### Bovine intestinal epithelial cell (BEC) and culture conditions

Bovine ileum was aseptically harvested from a neonatal Holstein calf that was euthanized under the approval of the University of Guelph Animal Care Committee. The ileum (15 cm) was flushed with tap water and placed in ice cold HBSS (Invitrogen) supplemented with 5x100 U/ml Penicillin/Streptomycin (Invitrogen), 2.5 μg/ml Fungizone (Invitrogen), and 25 μg/ml Gentamicin. After the tissue was washed with HBSS several times, the mucosa was scrapped off the underlying epithelium, which was then cut into small pieces, and washed three times in HBSS by centrifugation at 140 × g for 5 min at 4 °C to remove mucus. The tissue pellet (6–10 g) was then digested by shaking for 30 min in a 37 °C water bath in 30 ml of DMEM containing 150 U/ml collagenase I, 100 ~ 150 U/ml Penicillin/Streptomycin, 2.5 μg/ml Gentamicin and 2.5 μg/ml Fungizone. Following this, the epithelium was mechanically dispersed using a 25 ml serological pipette by drawing the digested tissue through it several times. DMEM containing 10 % FBS (approximately 30 ml) was added to stop enzymatic digestion, and the crypts were concentrated by centrifuged at 80 × g for 5 min. To isolate crypts, the cell pellet was diluted with DMEM and enriched 3–5 times by centrifugation over a 2 % D-sorbitol gradient for 5 min at 50 × g. The isolated crypts were washed with DMEM, and then cultured in the cell culture flask using DMEM supplemented with high glucose, 10 % heat inactivated FBS, 2.5 % HEPES buffer, 1 % Fungizone, 1 % Penicillin/Streptomycin, 1 % sodium pyruvate, 1 % insulin, 1 % non-essential amino acid solution, Gentamicin (25 μg/ml) and epidermal growth factor (30 ng/ml; EGF; Sigma-Aldrich); each crypt consists of 200–300 epithelial cells [27]. BECs were isolated as described by Kaushik et al. [28], with slight modification. Primary BECs were confirmed by fluorescent microscopy using the monoclonal anti-cytokeratin antibody pan (mixture) (Sigma-Aldrich), which reacts specifically with a wide variety of epithelial tissues, and the secondary antibody anti-mouse IgG FITC-conjugated antibody (Sigma-Aldrich). The primary BECs were subsequently frozen in cryopreservation media containing 10 % DMSO, 40 % DMEM and 50 % FBS at -80 °C for storage.

In preparation for the adhesion assay, frozen BECs were thawed at 37 °C and washed with DMEM centrifuged at 300 × g for 6 min. Following this, the pellet was re-suspended with DMEM supplemented with high glucose, 1 % heat inactivated FBS, 2.5 % HEPES buffer, 1 % Fungizone, 1 % Penicillin/Streptomycin, 1 % sodium pyruvate, 1 % insulin, 1 % non-essential amino acid solution, Gentamicin (25 μg/ml) and EGF (30 ng/ml) according to Bridger et al. [29], and cultured for at least a week to reach confluence.

### Assessment of epithelial cell viability

After reaching 80-100 % confluence in flask, epithelial cells were washed with warm phosphate buffered saline (PBS; Sigma-Aldrich), dislodged with TrypLE Express (Invitrogen) for 5 min, and counted with 0.4 % trypan blue (Invitrogen) using a hemocytometer chamber slide.

Cells were seeded into black 96-well flat bottom plates (50,000 cells per well) within cell culture medium that did not contain Fungizone and other antibiotics, and incubated at 37 **°**C with 5 % CO_2_ overnight (17 h for MAC-T cells and 21 h for BECs). Cells were then exposed to a range of concentrations (0.25, 0.5, 1,2, 4, 6, 8 and 16 mg/ml) of yeast CWCs from strains A, B, C and D of *S. cerevisiae* and two forms of dead yeast from strain A provided by Lallemand Inc., QC, Canada. The cell culture plate was centrifuged briefly at 200 × g for 3 min to ensure interaction between epithelial cells and yeast derivatives before incubation at 37 °C with 5 % CO_2_. In a preliminary trial, exposures of 2, 4, 6 and 24 h were assessed, and 6 h was deemed to be suitable when considering binding efficacy, epithelial cell proliferation and viability, and MAP proliferation. After a 6-h period, the epithelial cells were washed with warm PBS, then incubated with calcein AM diluted in culture media (Invitrogen) at room temperature for 30 min to stain live cells. The number of live cells was estimated by measuring the fluorescence of calcein AM (excitation 494/emission 517 nm) using a 1420 Victor2 Multilabel Counter (Beckman Coulter, Inc. California). The cell viability was calculated by using the formula: Percent of cell viability = (X/Y) × 100, where X is the fluorescence value in each well containing yeast derivative-treated cells and Y is the mean value of the fluorescence reading of all control wells.

### Assessment of yeast and CWC adhesive properties using MAP

The MAC-T cells/BECs were seeded into 96-black well flat bottom plates at 50,000 cells per well with cell culture medium that did not contain Fungizone and other antibiotics, and MAC-T cells and BECs were incubated for 17 and 21 h, respectively, at 37 °C with 5 % CO_2_. The cells were then exposed to a range of concentrations of yeast derivatives with MAP at a 10:1 (CFU: cell) multiplicity of infection. The cell culture plates were centrifuged at 200 × g for 3 min to ensure interaction between MAP and epithelial cells before incubation at 37 **°**C with 5 % CO_2_ for 6 h. Following this, the plate was washed with warm PBS, and the adhesion of MAP was estimated by measuring the fluorescence intensity of mCherry-MAP (excitation 587/emission 610 nm) using a 1420 Victor2 Multilabel Counter. The adhesion of MAP to epithelial cells was calculated using the formula: Adhesion of mCherry-MAP = the fluorescence value in each well of treatments with MAP infection (A) - the mean value of the fluorescence of all wells of the corresponding control group with same exposure concentration of yeast or yeast CWCs (B).

### Statistical analysis

The BEC and MAC-T cell viability were all analyzed separately as randomized complete block designs, in which the three independent trials represented the random blocks in each analysis. There were 6 replicates for each treatment within each block. The model for each of experiment included blocks as a random effect, and yeast derivatives and concentration level plus their interactions as fixed effects. All data were log-transformed prior to analysis in order to stabilize variances. Separate residual variances for each yeast derivative were incorporated in the model. Linear and quadratic orthogonal polynomial contrasts across concentration were used to assess changes in viability using the mixed procedure SAS 9.4 (SAS Institute Inc., 2012).

For the adhesion data, MAP adhesion to BECs or MAC-T cells was analyzed separately as a randomized complete block design with three independent trials representing each block. All data were log-transformed prior to analysis in order to stabilize variances. Concentration-dependent responses were compared between control and treatments by one-way ANOVA using Dunnett’s test for statistical significance with the mixed procedure from SAS 9.4 (SAS Institute Inc.).

Graphs were generated using the Graphpad Prism version 4.00 (GraphPad Software, 2003, San Diego California, USA), and all data were presented as least squares means of the log transformed data from the percentage of cell viability and adhesion of MAP. A *p*-value ≤ 0.05 was considered statistically significant.

## Results

### Effect of yeast derivatives on the viability of MAC-T cells and BEC

**Fig. 3:**
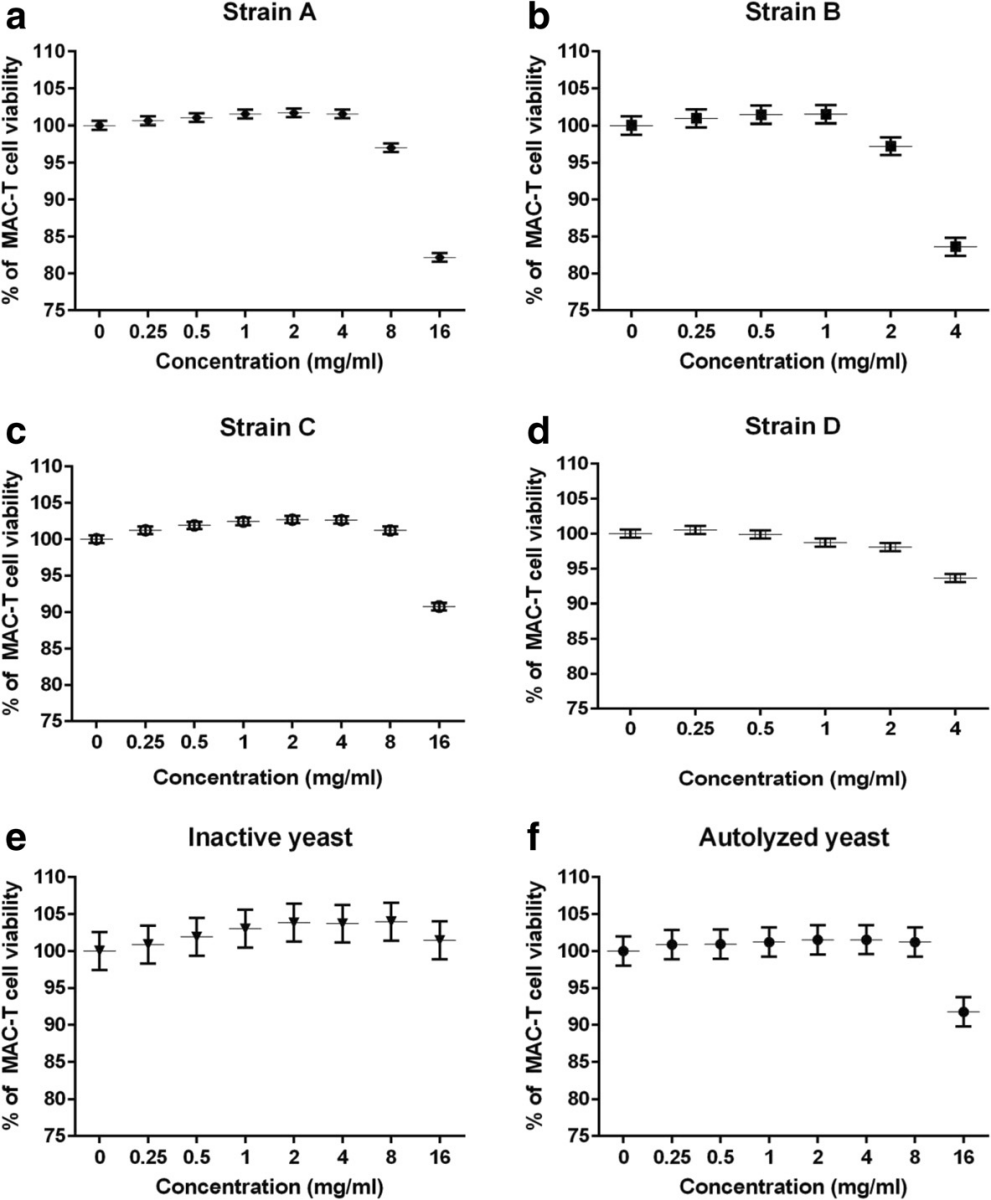
Viability of MAC-T cells following 6-h exposure to CWCs from yeast strain A (**a**), B (**b**), C (**c**) and D (**d**), and two forms of dead yeast from strain A (inactive yeast (**e**); autolyzed yeast (**f**)). Data are presented as least square mean +/- standard error

MAC-T cell viability was significantly reduced by CWCs from all strains as indicated by linear contrasts (*p* < 0.01, Fig. 3a-d). In contrast, MAC-T cell viability was significantly increased by inactive yeast from strain A (*p* < 0.01) as indicated by quadratic contrasts, and no cytotoxicity was observed (Fig. 3e). The autolyzed yeast also reduced MAC-T viability at the highest concentration (16 mg/ml) and this resulted in a significant linear contrast across concentration (*p* < 0.01, Fig. 3f).

**Fig. 4:**
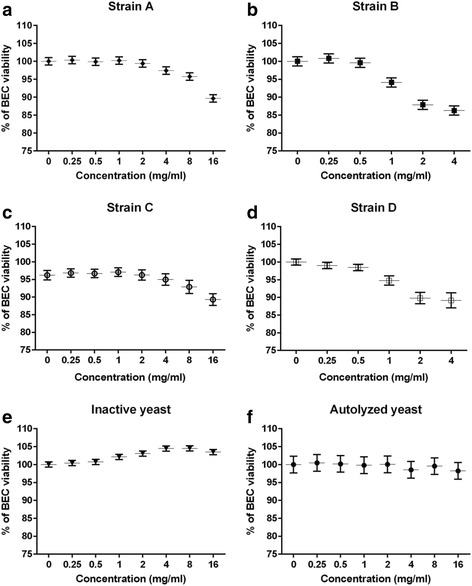
Viability of BECs following 6-h exposure to CWCs from yeast strain A (**a**), B (**b**), C (**c**) and D (**d**), and two forms of dead yeast from strain A (inactive yeast (**e**); autolyzed yeast (**f**)). Data are presented as least square mean +/- standard error

BEC viability was significantly reduced by CWCs from all strains as indicated by linear contrasts (*p* < 0.01, Fig. 4a-d). BEC viability was also significantly increased by inactive yeast from strain A as indicated by quadratic contrasts, and cytotoxicity was not observed for either form of dead yeast from strain A (*p* < 0.01, Fig. 4e-f).

### Effect of yeast derivatives on MAP binding to MAC-T cell and BEC

**Fig. 5:**
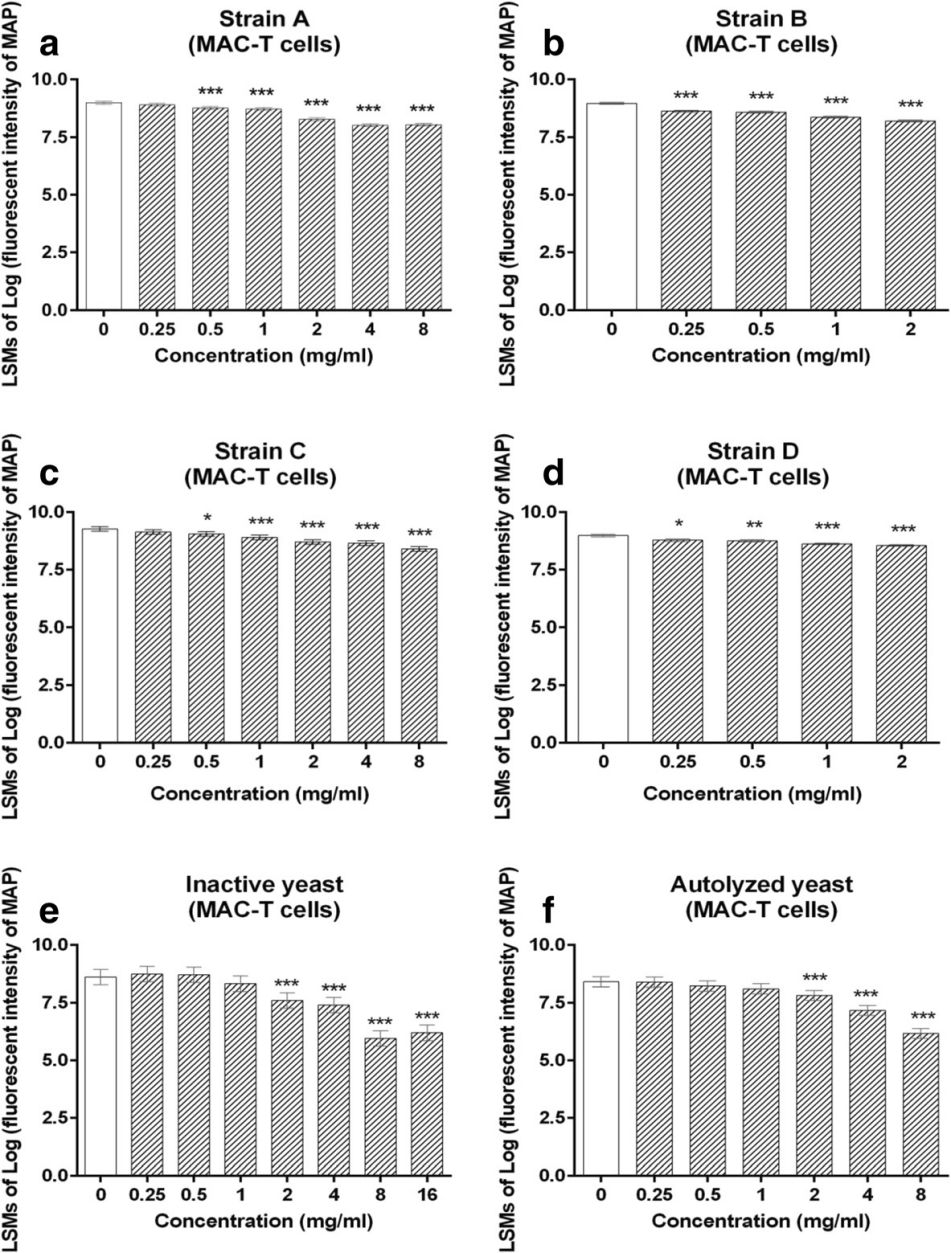
Binding of mCherry-MAP to MAC-T cells in the presence of yeast CWCs from yeast strain A (**a**), B (**b**), C (**c**) and D (**d**), and two forms of dead yeast from strain A (inactive yeast (**e**); autolyzed yeast (**f**)) after 6-h exposure. Data are presented as least square mean +/- standard error. Significant differences relative to the control are indicated at *p* < 0.05 (*), 0.01 (**), and 0.001 (***)

CWCs from the four yeast strains concentration-dependently reduced MAP binding to MAC-T cells under cultured conditions for 6 h. There was significant reduction in the number of MAP adherent to MAC-T cells after 6-h exposure to CWCs from strain A and C at 0.5–8 mg/ml (*p* < 0.05, Fig. 5a and 5c). A significant reduction of MAP adhesion was also seen when MAC-T cells were cultured with all concentrations of CWCs from strain B and D (*p* < 0.05, Fig. 5b and 5d). Similarly, the number of MAP adhering to MAC-T cells appeared to decrease as the concentration of both forms of dead yeast from strain A increased; the significant reduction can be seen from concentrations 2 mg/ml and higher for both inactive and autolyzed yeast (*p* < 0.05, Fig. 5e-f).

**Fig. 6:**
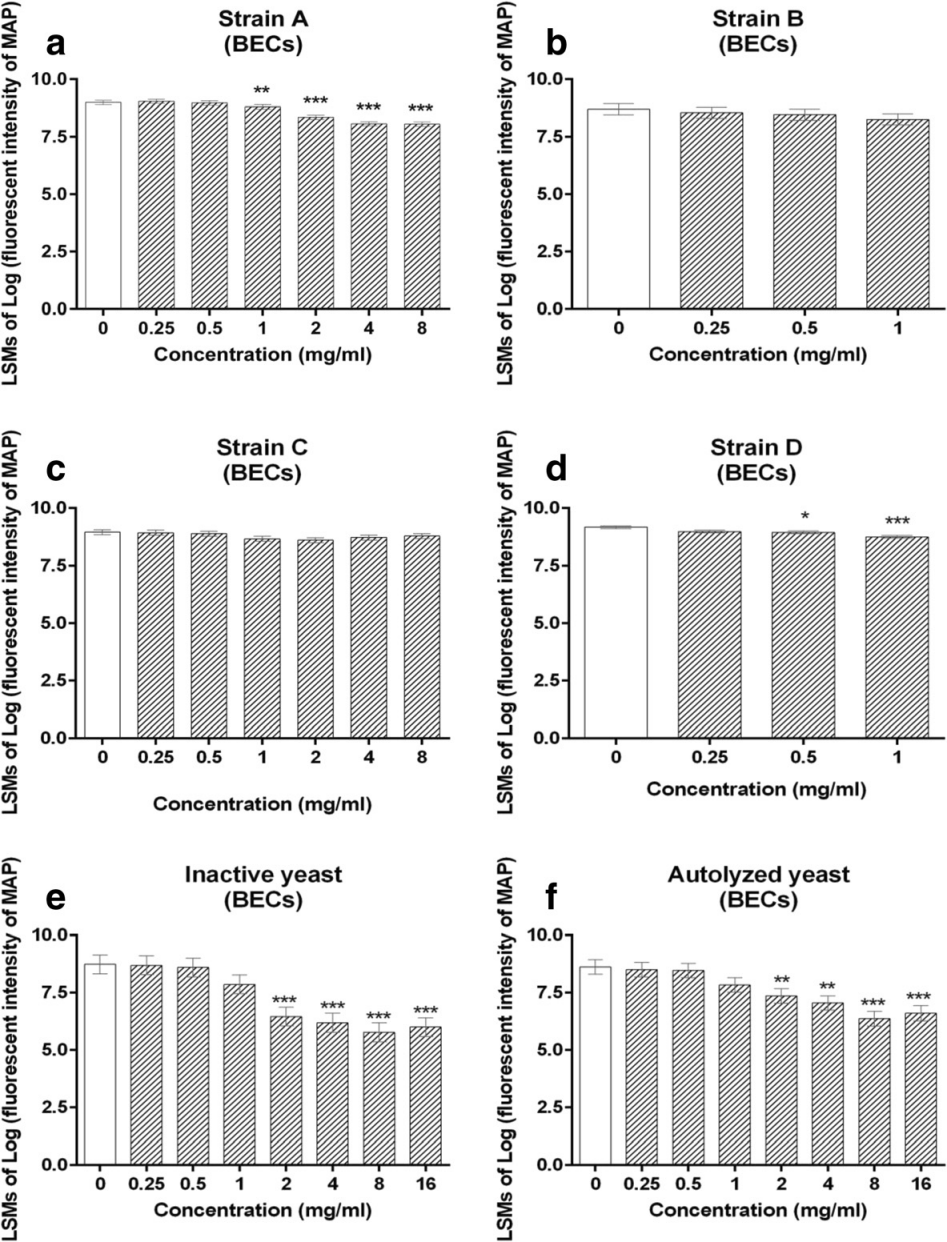
Binding of mCherry-MAP to BECs in the presence of yeast CWCs from yeast strain A (**a**), B (**b**), C (**c**) and D (**d**), and two forms of dead yeast from strain A (inactive yeast (**e**); autolyzed yeast (**f**)) after 6-h exposure. Data are presented as least square mean +/- standard error. Significant differences relative to the control are indicated at *p* < 0.05 (*), 0.01 (**), and 0.001 (***)

CWCs from strain A and D also concentration-dependently reduced MAP binding to BECs during the 6-h exposure period. A significant reduction in MAP binding was observed from strain A at concentrations 1-8 mg/ml, and from strain D at concentrations 0.5-1 mg/ml (*p* < 0.05, Fig. 6a and 6d). However, there was no significant difference in MAP binding in response to CWCs from strain B and C (Fig. 6b and 6c). Additionally, a concentration-dependent reduction of MAP binding to BECs was observed with both inactive and autolyzed yeast from strain A, with significant differences appearing between 2-16 mg/ml (*p* < 0.05, Fig. 6e-f).

## Discussion

In this study, the adherence of MAP to bovine epithelial cells was significantly reduced in cell culture by the addition of yeast *S. cerevisiae* and its’ CWCs. In terms of effectiveness, the two forms of dead yeast from strain A had the most significant impact on preventing MAP adhesion to MAC-T cells and BECs, with maximal binding for the autolyzed and inactive yeast being around 26 and 30 %, respectively. To our knowledge, this is the first investigation of the adhesive properties of *S. cerevisiae* components against MAP. The MAC-T cells were used in this study because of ease of culture and lack of a suitable bovine small intestinal epithelial cell line, and it is challenging to obtain sufficient numbers of primary intestinal epithelial cells for high through-put *in vitro* screening of bioactive compounds. One concern with using MAC-T cells is that they may not appropriately model MAP binding to intestinal epithelial cells; however, in this study MAP adhesion appeared similar between MAC-T cells and primary BECs. Additionally, MAP has also previously been detected within mammary gland of sub-clinically infected cows [30], which suggests that MAP is able to disseminate and invade the mammary epithelium.

Another potential concern with assessing adhesion of yeast and its CWCs to MAP under cell culture conditions is that it may influence epithelial cell viability. The viability of both MAC-T cells and BECs was therefore tested after a 6-h exposure period with the yeast and its CWCs. The results varied with the epithelial cell type and yeast component. MAC-T cell viability for example, appeared to be lower than that of BECs within the same treatment of CWCs from strain A at 16 mg/ml, and the autolyzed yeast from strain A at 16 mg/ml. The decrease of cell viability might be related to changes in intracellular homeostasis and apoptosis-regulated gene expression. Higher concentrations of yeast derivatives for example, may induce cell apoptosis via altering intracellular calcium levels and the ratio of Bax and bcl-2 genes, as was demonstrated in human breast cancer cells exposed to heat-killed *S. cerevisiae* [31]. Bax and bcl-2 are two discrete members of a gene family involved in the regulation of cellular apoptosis; Bax protein is known as an apoptosis-promoting factor, whereas bcl-2 protein as an apoptosis-suppressing factor [32–34]. Future studies will explore whether or not yeast components from this study induced epithelial cell apoptosis.

It was interesting to note that not all of the yeast components assessed in this study reduced the cell viability. Inactive yeast for example, actually increased the viability of both MAC-T cells and BECs in a quadratic manner. It is possible that inactive yeast stimulated cell proliferation during the 6-h exposure period. Increased cell proliferation was observed with lymphocytes isolated from weaned pigs [35]; in this study, lymphocytes were stimulated with different concentrations of β-glucans from a variety of sources, and *S. cerevisiae* β-glucan significantly induced the cell proliferation compared with the negative control.

Another potential concern about the following observations is that the reduction of MAP binding to epithelial cells was partially attributed to a decrease of epithelial cell viability. CWCs from strain B for instance, reduced MAC-T cell viability and MAP binding at the same concentrations (2 and 4 mg/ml); thus, the reduction of MAP binding could have, in part, been attributed to a decrease in the number of viable epithelial cells. However, a reduction in MAP binding to epithelial cells was also observed at lower non-cytotoxic concentration of CWC from strain B and with the two forms of dead yeast from strain A without a decrease of cell viability.

In addition to the observed decrease in MAP binding to epithelial cells in the presence of *S. cerevisiae* components, reduced MAP infection has also been demonstrated with mice fed the probiotic *Lactobacillus animalis* [36]. Thus, both studies provide evidence that risk of MAP infection may be reduced in ruminants by probiotic/prebiotic use. Similar observations have also been observed with other enteropathgenic bacteria such as *E. coli, Salmonella* Typhimurium and *Salmonella* Typhi when *S. cerevisiae* was used as an adhesive agent [37–39]. Although these later studies were carried out in vivo, making comparisons with the present study difficult, these studies and our study suggest that *S. cerevisiae* may be used, in milk replacer for example, to reduce calf exposure to Gram-negative pathogens and MAP. This hypothesis should be validated in vivo, since increased yeast binding to MAP may not necessarily translate to lower infection load. Ganan et al. [10] for example, demonstrated that while whole yeast, yeast CWC, and the mannose fraction blocked adherence of *Campylobacter jejuni* to Caco-2 cells by approximately 55, 85 and 55 %, respectively, only the mannose fraction effectively reduced microbial invasion. In the present study, the whole cell lysate (inactive yeast and autolyzed yeast) were much more effective at inhibiting MAP binding to MAC-T and BECs than CWCs, and at higher concentrations (>2 mg/ml), the adherence was reduced by around 10–30 % in the presence of inactive yeast (Figs. 5e and 6e). Differences in binding efficacy between the Ganan et al. and present study are likely attributed to the different components within the *C. jejuni* and MAP cell membranes. MAP for example, has a much thicker cell wall than Gram-negative species (*C. jejuni*), and it is made up of a hydrophobic outer mycolate layer and inner peptidoglycan layer bound together by the polysaccharide, arabinogalactan [40]. In contrast, the cell membrane of Gram-negative species is made up of an outer lipopolysaccharide layer and a thin inner peptidoglycan layer [41].

The present in vitro binding assay that involved mCherry-MAP and bovine epithelial cells offers a potentially high through-put platform for screening the adhesive properties of probiotics/prebiotics and their bioactive components that may be useful for controlling JD. Another potential concern about this in vitro study, however, is that the yeast components may influence other mucosal cell types such as goblet cells and dendritic cells (DCs) that were not taken into consideration with this cell culture model. For example, in previous in vivo studies, dietary supplementation with MOS derived from *S. cerevisiae* increased the number of cells secreting acid mucins in posterior gut of sea bass [42]. Increased mucus secretion induced by MOS may help prevent pathogenic bacteria adhesion via the anti-adhesive action of mucins [43], as well as via the bulk physical properties of mucus that help to clear bacteria [44]. However, a reduction in the number of goblet cells that produce mucus has also been reported in the ileal sections of chicken poults fed 0.02 % *S. cerevisiae var boulardii* [45].

Yeast components can also have interactions with DCs. Mannan derived from the *S. cerevisiae* or *Candida albicans* cell wall for example, has been reported to induce the maturation of murine DCs *in vivo* [46]. DC maturation is required for the priming of T helper 1 (Th1) or T helper 2 (Th2) cells, both of which are involved in the adaptive immune response. Sheng et al. [46] also showed that two different types of mannan, oxidized mannan and reduced mannan, differentially stimulated Th1/Th2 cytokine production from murine bone-marrow-derived DCs. Th1 cytokines play a key role during the cell-mediated immune response, which is required to control MAP infection [47]; whereas, the stimulation of Th2 cytokines that support an antibody-mediated immune response is thought to be insufficient for controlling intracellular pathogens such as MAP [48]. Given the potential limitation of the present binding assay with using mCherry-MAP, *in vivo* studies are also needed to validate the benefits of promising yeast and yeast CWCs that have been identified through *in vitro* screening.

In addition to influencing host mucosal cells, yeast probiotics and their CWCs may also influence commensal bacteria and protozoa that reside within ruminant gastrointestinal tract. It was reported that MOS treatment for example, significantly increased the diversity and band number of bacteria in both ileal and colonic fermentum from piglets [49], which implies that MOS could stabilize the gastrointestinal tract microflora that may act as a protective barrier against MAP infection. Additionally, feeding yeast culture was previously reported to increase the number of protozoa in the rumen [50], since the yeast culture is used as a protein and energy source by protozoa [51, 52]. Given that two commonly occurring environmental protozoa, *Acanthamoeba castellanii and A. polyphaga*, have been reported to be the vectors for MAP [53], it is still remains to be determined if the oral administration of yeast or yeast CWCs will help reduce risk of MAP infection.

## Conclusions

The results from the present study demonstrate that the adhesion of MAP to epithelial cells was significantly reduced *in vitro* by *S. cerevisiae* and its’ CWC in a strain-dependent manner. Since MAP is an intracellular pathogen, this anti-adhesive action could potentially reduce uptake of MAP by epithelial cells. However, since the specific mechanism of reduced MAP adhesion remains to be elucidated, further characterization and investigation is warranted to determine potential mechanisms of action, the stability of the binding between yeast components and MAP, and the efficacy of the tested yeast components *in vivo*.

## Abbreviations

BEC: Bovine primary intestinal epithelial cell
CFU: Colony forming units
CWC: Cell wall component
JD: Johne’s disease
MAC-T cell: Bovine mammary epithelial cell line
MAP: *Mycobacterium avium* subspecies *paratuberculosis*
MOS: Mannan oligosaccharides

## References

[1] Williams PEV, Tait CA, Innes GM, Newbold CJ (1991). Effects of the inclusion of yeast culture (*Saccharomyces cerevisiae* plus growth medium) in the diet of dairy cows on milk yield and forage degradation and fermentation patterns in the rumen of steers. J Anim Sci.

[2] Robinson PH, Garrett JE (1999). Effect of yeast culture (*Saccharomyces cerevisiae*) on adaptation of cows to postpartum diets and on lactational performance. J Anim Sci.

[3] Salama AAK, Caja G, Garín D, Albanell E, Such X, Casals R (2002). Effects of adding a mixture of malate and yeast culture (*Saccharomyces cerevisiae*) on milk production of Murciano-Granadina dairy goats. Anim Res..

[4] Wohlt JE, Corcione TT, Zajac PK (1998). Effect of yeast on feed intake and performance of cows fed diets based on corn silage during early lactation. J Dairy Sci..

[5] Kamel HEM, Sekine J, El-Waziry AM, Yacout MHM (2004). Effect of *Saccharomyces cerevisiae* on the synchronization of organic matter and nitrogen degradation kinetics and microbial nitrogen synthesis in sheep fed Berseem hay (*Trifolium alexandrinum*). Small Rumin Res..

[6] Doreau M, Jouany JP (1998). Effect of a *Saccharomyces cerevisiae* culture on nutrient digestion in lactating dairy cows. J Dairy Sci..

[7] Jouany JP, Mathieu F, Senaud J, Bohatier J, Bertin G, Mercier M (1998). The effect of *Saccharomyces cerevisiae* and *Aspergillus oryzae* on the digestion of the cell wall fraction of a mixed diet in defaunated and refaunated sheep rumen. Reprod Nutr Dev..

[8] Mosoni P, Chaucheyras-Durand F, Béra-Maillet C, Forano E (2007). Quantification by real-time PCR of cellulolytic bacteria in the rumen of sheep after supplementation of a forage diet with readily fermentable carbohydrates: effect of a yeast additive. J Appl Microbiol..

[9] Silberberg M, Chaucheyras-Durand F, Mialon MM, Monteils V, Mosoni P, Morgavi DP, Martin C (2013). Repeated acidosis challenges and live yeast supplementation shape rumen microbiota and fermentations and modulate inflammatory status in sheep. Animal..

[10] Ganan M, Carrascosa AV, de Pascual-Teresa S, Martinez-Rodriguez AJ (2009). Inhibition by yeast-derived mannoproteins of adherence to and invasion of Caco-2 cells by *Campylobacter jejuni*. J Food Prot.

[11] Baurhoo B, Letellier A, Zhao X, Ruiz-Feria CA (2007). Cecal populations of lactobacilli and bifidobacteria and Escherichia coli populations after in vivo Escherichia coli challenge in birds fed diets with purified lignin or mannanoligosaccharides. Poult Sci.

[12] Fernandez F, Hinton M, Gils BV (2002). Dietary mannan-oligosaccharides and their effect on chicken caecal microflora in relation to Salmonella Enteritidis colonization. Avian Pathol.

[13] Posadas SJ, Caz V, Caballero I, Cendejas E, Quilez I, Largo C, Elvira M, De Miguel E (2010). Effects of mannoprotein E1 in liquid diet on inflammatory response and TLR5 expression in the gut of rats infected by *Salmonella typhimurium*. BMC Gastroenterol.

[14] Ganner A, Stoiber C, Wieder D, Schatzmayr G (2010). Quantitative in vitro assay to evaluate the capability of yeast cell wall fractions from *Trichosporon mycotoxinivorans* to selectively bind Gram-negative pathogens. J Microbiol Methods.

[15] Hines ME, Kreeger JM, Herron AJ (1995). Mycobacterial infections of animals: pathology and pathogenesis. Lab Anim Sci.

[16] Veterinary Laboratories Agency (VLA) (2008). Surveillance Report. Johne’s disease continues to be the most common cause of bovine enteric disease. Vet Rec..

[17] Windsor PA, Whittington RJ (2010). Evidence for age susceptibility of cattle to Johne’s disease. Vet J.

[18] Whitlock RH, Buergelt C (1996). Preclinical and clinical manifestations of paratuberculosis (including pathology). Vet Clin North Am Food Anim Pract.

[19] Coussens PM (2001). *Mycobacterium paratuberculosis* and the bovine immune system. Anim Health Res Rev.

[20] Duffield BJ, Young DA (1985). Survival of *Mycobacterium bovis* in defined environmental conditions. Vet Microbiol..

[21] Desikan KV (1995). Extended studies on the viability of *Mycobacterium leprae* outside the human body. Lepr Rev..

[22] Jackson R, Morris RS (1995). A study of the environmental survival of Mycobacterium bovis on a farm in New Zealand. New Zealand Vet J.

[23] Rosseels V, Huygen K (2008). Vaccination against paratuberculosis. Exp Rev Vaccines.

[24] National Center for Animal Health Programs-APHIS. Johne’s disease in cattle. http://www.aphis.usda.gov/publications/animal_health/content/printable_version/faq_johnes_disease08.pdf (2008). Accessed Nov 2008.

[25] Q. You, P, Mead, S. Oh, S. Mallikarjunappa, L. Mutheria, and N.A. Karrow 2014. Construction of a reporter Mycobacterium avium subsp. paratuberculosis (Map) strain and infection of monocyte-derived macrophages from cows homozygous for SNP -298 A > G in the macrophage migration inhibitory factor (MIF) gene. Proceedings of the 10th World Congress on Genetics Applied to Livestock Production. P 541. Vancouver, B.C. August 17–22.

[26] Huynh HT, Robitaille G, Turner JD (1991). Establishment of bovine mammary epithelial cells (MAC-T): an in vitro model for bovine lactation. Exp Cell Res.

[27] Föllmann W, Weber S, Birkner S (2000). Primary cell cultures of bovine colon epithelium: isolation and cell culture of colonocytes. Toxicol In Vitro.

[28] Kaushik RS, Begg AA, Wilson HL, Aich P, Abrahamsen MS, Potter A (2008). Establishment of fetal bovine intestinal epithelial cell cultures susceptible to bovine rotavirus infection. J Virol Methods.

[29] Bridger PS, Mohr M, Stamm I, Fröhlich J, Föllmann W, Birkner S (2010). Primary bovine colonic cells: a model to study strain-specific responses to *Escherichia coli*. Vet Immunol Immunopathol.

[30] Streeter RN, Hoffsis GF, Bech-Nielsen S, Shulaw WP, Rings DM (1995). Isolation of *Mycobacterium paratuberculosis* from colostrum and milk of subclinically infected cows. Am J Vet Res.

[31] Ghoneum M, Matsuura M, Braga M, Gollapudi S (2008). *S. cerevisiae* induces apoptosis in human metastatic breast cancer cells by altering intracellular Ca2+ and the ratio of Bax and Bcl-2. Int J Oncol.

[32] Oltval ZN, Milliman CL, Korsmeyer SJ (1993). Bcl-2 heterodimerizes in vivo with a conserved homolog, Bax, that accelerates programed cell death. Cell.

[33] Hockenbery D, Nuñez G, Milliman C, Schreiber RD, Korsmeyer SJ (1990). Bcl-2 is an inner mitochondrial membrane protein that blocks programmed cell death. Nature.

[34] Yang E, Korsmeyer SJ (1996). Molecular thanatopsis: a discourse on the BCL2 family and cell death. Blood..

[35] Sonck E, Stuyven E, Goddeeris B, Cox E (2010). The effect of beta-glucans on porcine leukocytes. Vet Immunol Immunopathol.

[36] Karunasena E, Kurkure PC, Lackey RD, McMahon KW, Kiernan EP, Graham S, Alabady MS, Campos DL, Tatum OL, Brashears MM (2013). Effects of the probiotic *Lactobacillus animalis* in murine *Mycobacterium avium* subspecies *paratuberculosis* infection. BMC Microbiol..

[37] Tiago FC, Martins FS, Souza EL, Pimenta PF, Araujo HR, Castro IM, Brandão RL, Nicoli JR (2012). Adhesion to the yeast cell surface as a mechanism for trapping pathogenic bacteria by *Saccharomyces* probiotics. J Med Microbiol.

[38] Becker PM, Galletti S (2008). Food and feed components for gut health-promoting adhesion of *E. coli* and *Salmonella enterica*. J Sci Food Agric.

[39] Chaucheyras-Durand F, Ossa F, Habouzit C, Castex M, Henri D. Effect of live yeast and yeast derivatives on adhesion capacity of *E.coli* strains to intestinal epithelial cells. In: Symposium on Gut Health in Production of Food Animals, College Station, Texas, US. 2012. https://ca.linkedin.com/in/faisuryossa. Accessed 3–5 Dec 2012.

[40] Ghuysen JM, Hakenbeck R (1994). Bacteria Cell Wall.

[41] Archibald AR, Hancock IC, Harwood CR (1993). Bacillus subtilis and other gram-positive bacteria: biochemistry, physiology, and molecular genetics.

[42] Torrecillas S, Makol A, Caballero MJ, Montero D, Ginés R, Sweetman J, Izquierdo M (2011). Improved feed utilization, intestinal mucus production and immune parameters in sea bass (Dicentrarchus labrax) fed mannan oligosaccharides (MOS). Aquac Nutr.

[43] Carlstedt I, Davies JR (1997). Glycoconjugates facing the outside world. Biochem Soc Trans.

[44] Sandberg T, Nestor M, Pahlson C, Shi L, Caldwell KD. Mucin as surface protectant against bacterial adhesion. In: Abstract of papers of the American Chemical Society. Amer Chemical Soc. 2000. http://uu.diva-portal.org/smash/record.jsf?pid=diva2%3A66296&dswid=-5673. Accessed 17 Oct 2008

[45] Bradley GL, Savage TF, Timm KI (1994). The effects of supplementing diets with *Saccharomyces cerevisiae* var. *boulardii* on male poult performance and ileal morphology. Poult Sci.

[46] Sheng KC, Pouniotis DS, Wright MD, Tang CK, Lazoura E, Pietersz GA, Apostolopoulos V (2006). Mannan derivatives induce phenotypic and functional maturation of mouse dendritic cells. Immunology.

[47] Pieters J (2001). Entry and survival of pathogenic mycobacteriain macrophages. Microbes Infect.

[48] Alonso-Hearn M, Patel D, Danelishvili L, Meunier-Goddik L, Bermudez LE (2008). The *Mycobacterium avium* subsp. *paratuberculosis* MAP3464 gene encodes an oxidoreductase involved in invasion of bovine epithelial cells through the activation of host cell Cdc42. Infect Immun.

[49] Hang S, Zhu W (2012). Gut bacterial and Lactobacilli communities of weaning piglets in response to mannan oligosaccharide and sugar beet pulp in vitro fermentation. J. Integr. Agr..

[50] Arakaki LC, Stahringer RC, Garrett JE, Dehority BA (2000). The effects of feeding monensin and yeast culture, alone or in combination, on the concentration and genetic composition of rumen protozoa in steers fed on low-quality pasture supplemented with increasing levels of concentrate. Anim Feed Sci Technol.

[51] Dehority BA (1986). Protozoa of the digestive tract of herbivorous mammals. Int J Trop Insect Sci.

[52] Dehority BA, Orpin CG, Hobson PN, Stewart CS (1997). Development of, and natural fluctuations in, rumen microbial populations. The Rumen Microbial Ecosystem.

[53] Whan L, Grant IR, Rowe MT (2006). Interaction between Mycobacterium avium subsp. paratuberculosis and environmental protozoa. BMC Microbiol.

